# Metalloporphyrin‐Based Cathode for Rechargeable Magnesium‐Ion Batteries: Copper Leaching and Interphase Formation

**DOI:** 10.1002/cssc.202501463

**Published:** 2025-11-11

**Authors:** Tom Philipp, Riccarda Müller, Till Ortmann, Christine Kern, Marcus Rohnke, Simon Schauer, Mika Lindén, Ebrahim Abouzari‐Lotf, Thomas Smok, Maximilian Fichtner, Shirin Shakouri, Mario Ruben, Kerstin Leopold, Christine Kranz

**Affiliations:** ^1^ Institute of Analytical and Bioanalytical Chemistry (IABC) Ulm University Albert‐Einstein‐Allee 11 89081 Ulm Germany; ^2^ Institute of Physical Chemistry and Center for Materials Research Justus Liebig University Giessen Heinrich‐Buff‐Ring 17 35392 Giessen Germany; ^3^ Institute of Inorganic Chemistry II Ulm University Albert‐Einstein‐Allee 11 89081 Ulm Germany; ^4^ Solid State Chemistry Helmholtz Institute Ulm (HIU) Helmholtzstraße 11 89081 Ulm Germany; ^5^ Institute of Nanotechnology (INT) Karlsruhe Institute of Technology (KIT) Hermann‐von‐Helmholtz‐Platz 1 76344 Eggenstein‐Leopoldshafen Germany; ^6^ Institute of Quantum Materials and Technologies (IQMT) Karlsruhe Institute of Technology (KIT) Kaiserstraße 12 76131 Karlsruhe Germany; ^7^ Centre Européen de Sciences Quantiques (CESQ) Institut de Science et d’Ingénierie Supramoléculaires (ISIS) 8 allée Gaspard Monge, BP 70028 67083 Strasbourg Cedex France

**Keywords:** copper leaching, Cu‐porphyrin, osmium tetroxide staining, rechargeable magnesium‐ion batteries, transmetalation

## Abstract

[5,15‐bis(ethynyl)‐10,20‐diphenylporphinato]copper(II) (CuDEPP) composite cathodes for rechargeable magnesium‐ion batteries have been investigated before, after, and during initial cycling (pristine, first charging, and consecutive discharging). The initial self‐conditioning is studied by scanning electron microscopy and spectroscopy‐based methods (energy‐dispersive X‐ray spectroscopy, total reflection X‐ray fluorescence spectrometry, and Raman spectroscopy) as well as time‐of‐flight secondary ion mass spectrometry (ToF‐SIMS) and orbitrap SIMS (Orbi‐SIMS). This study shows that copper is released from the active material into the electrolyte solution during the initial cycling at potentials higher than 3.1 V versus Mg^2+^/Mg. The results point toward a transmetalation process transforming CuDEPP into [5,15‐bis(ethynyl)‐10,20‐diphenylporphinato]magnesium(II) (MgDEPP). Further, the self‐conditioning process *via* electro‐polymerization observed for monovalent ions appears to be absent in the investigated Mg‐based system, as the alkynyl group of the CuDEPP seems to remain unaltered during the initial charging and discharging steps. Additionally, cross‐sectional and laterally resolved ToF‐SIMS image analyses show an accumulation of inorganic fluorine‐rich copper and magnesium species at the electrode surface including the active material exposed to the electrolyte after cycling that might be a result of a passivation layer or the formation of a cathodic electrolyte interphase.

## Graphical abstract

The initial cycling of [5,15‐bis(ethynyl)‐10,20‐diphenylporphinato]copper(II) electrodes in rechargeable Mg‐ion batteries is studied using electrochemical, spectroscopic, microscopic, and spectrometric methods such as total reflection X‐ray fluorescence spectrometry and time‐of‐flight secondary ion mass spectrometry . Copper dissolution above 3.1 V versus Mg^2+^/Mg indicates transmetalation. ToF‐SIMS imaging reveals fluorine‐rich copper and magnesium species at the surface, suggesting passivation or cathodic electrolyte interphase formation.

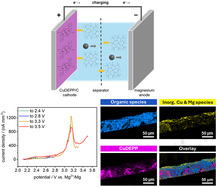

## 1 Introduction

**Figure 1:**
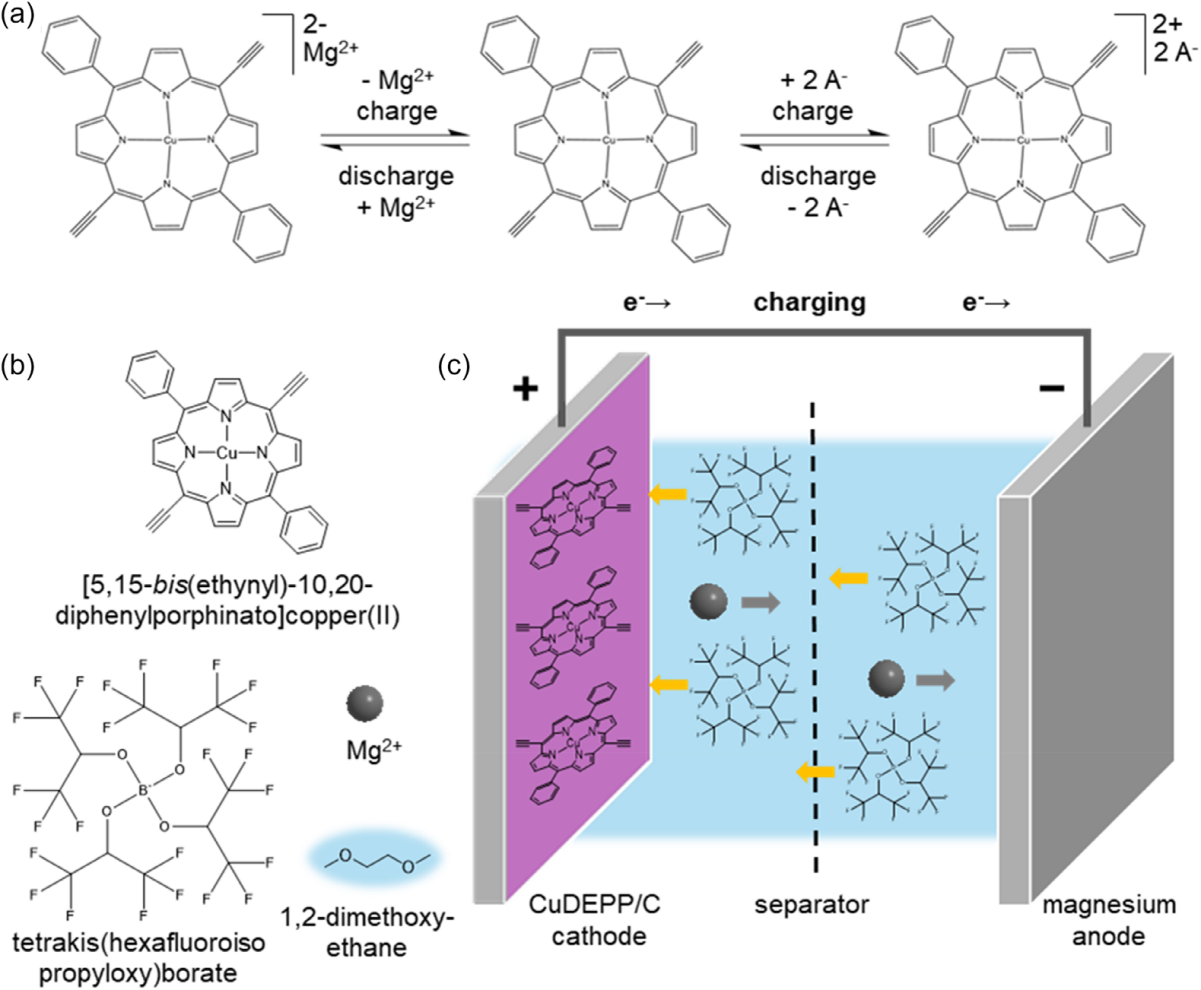
a) General depiction of the two‐electron reduction and oxidation displaying the bipolar behavior of the 18*π*‐electron system of [5,15‐bis(ethynyl)‐10,20‐diphenylporphinato]copper(II) (CuDEPP). b) Schematic representation of CuDEPP and the electrolyte components magnesium tetrakis(hexafluoroisopropyloxy)borate (Mg[B(hfip)_4_]_2_) in 1,2‐dimethoxyethane (DME). c) The cell configuration for a rechargeable Mg‐ion half‐cell using CuDEPP as the active cathode material and Mg metal as the anode.

The increase in energy demand requires a diversification in energy generation and in energy storage.^[^ ^1^ ^]^ Additionally, new technologies in energy storage and battery development must satisfy the demands of the economy, but also contribute to a better sustainability.^[^ ^2^ ^]^ Organic electrode materials can be environmentally friendly when synthesized from abundant precursors and thereby minimize the environmental impact in comparison to their inorganic counterparts presently used.^[^ ^3^, ^4^ ^]^ Due to their functionality and wide variety, organic electrode materials offer a plethora of different reaction mechanisms for energy storage processes.^[^ ^5^ ^]^ Different organic material classes, e.g., organic disulfides,^[^ ^6^ ^]^ carbonyl compounds,^[^ ^7^, ^8^ ^]^ polymers,^[^ ^9^, ^10^ ^]^ and metalloorganic coordination complexes,^[^ ^11^ ^]^ have been investigated. Organic materials face challenges such as low conductivity, sluggish ion diffusion, and electrolyte instability. Further, they are limited by dissolution and anode interface issues. Recent advances focus on molecular design and electrolyte optimization to improve cycling stability and rate performance while overcoming these limitations.^[^ ^12^, ^13^, ^14^ ^]^ Among the metalloorganic coordination compounds, porphyrin‐based materials have been gaining increased attention in the field of energy storage and conversion during recent years.^[^ ^15^, ^16^, ^17^, ^18^ ^]^ Especially, the bipolar behavior of the conjugated 18*π*‐electron system, which enables a two‐electron reduction and oxidation of the material (**Figure** **1**a), makes porphyrins attractive as electrode materials in dual‐ion batteries.^[^ ^19^ ^]^ With different substituents or central metal atoms, porphyrin‐based materials have been investigated as cathode materials in lithium‐ion batteries (LIBs),^[^ ^20^, ^21^, ^22^, ^23^, ^24^, ^25^, ^26^, ^27^ ^]^ sodium‐ion batteries (SIBs),^[^ ^20^, ^28^, ^29^ ^]^ potassium‐ion batteries (KIBs),^[^ ^20^ ^]^ as well as in rechargeable calcium batteries^[^ ^30^ ^]^ and aluminum batteries.^[^ ^16^, ^31^, ^32^ ^]^

The metalloporphyrin complex [5,15‐bis(ethynyl)‐10,20‐diphenylporphinato]copper(II) (CuDEPP) has been explored as a cathode material in LIBs,^[^ ^23^, ^24^, ^25^, ^26^, ^27^ ^]^ SIBs,^[^ ^33^ ^]^ KIBs,^[^ ^34^ ^]^ as well as in rechargeable magnesium batteries (RMBs),^[^ ^35^ ^]^ achieving good theoretical capacities up to 187 mAh g^−1^ and a high capacity retention of 85% for 2000 cycles in the case of LIBs.^[^ ^23^ ^]^ Furthermore, fast charge and discharge rates up to 53 °C,^[^ ^23^ ^]^ due to its pseudocapacitive behavior, may allow the use in high‐power applications.

When CuDEPP is used for monovalent ion (Li^+^, Na^+^, and K^+^) batteries, CuDEPP undergoes a self‐conditioning step^[^ ^23^, ^33^, ^34^, ^36^ ^]^ during the first charge cycle, resulting in a highly reversible and stable charge–discharge process.^[^ ^23^ ^]^ This initial conditioning step has been the subject of several studies using, e.g., powder X‐ray diffraction (XRD) and infrared spectroscopy,^[^ ^23^ ^]^ scanning electron microscopy (SEM) combined with energy‐ and wavelength‐dispersive X‐ray spectroscopy (EDX/WDX) and Raman spectroscopy.^[^ ^24^ ^]^ During the conditioning process, the ethynyl substituents undergo a chemical change, presumably due to an electrochemically induced polymerization, leading to an extended *π*‐conjugated network. This reduces the charge transfer resistance, as shown by electrochemical impedance spectroscopy.^[^ ^23^ ^]^

Further, Raman measurements revealed a distortion within the porphyrin‐substructure,^[^ ^24^ ^]^ probably related to the insertion of electrolyte ions leading to a loss in crystallinity as determined by XRD^[^ ^23^ ^]^ and electron diffraction.^[^ ^24^ ^]^ For bivalent ions, e.g., Mg^2+^, the self‐conditioning process remains ambiguous and has not yet been fully clarified. It is assumed that there is no electrochemically induced polymerization *via* the ethynyl moiety and that the crystalline structure of the active material is less affected by the distortion of the lattice due to ion insertion during cycling.^[^ ^35^ ^]^ Additionally, UV/vis spectroscopy pointed toward a transmetalation process from CuDEPP to MgDEPP or an intermediate metal‐free species. This finding is supported by XPS analysis.^[^ ^35^ ^]^

In this study, we present further proof that there is a transmetalation step during the initial self‐conditioning process. We investigated copper (Cu) leaching from the CuDEPP composite cathodes in half‐cells versus magnesium (Mg) metal in magnesium tetrakis(hexafluoroisopropyloxy)borate in 1,2‐dimethoxyethane electrolyte^[^ ^37^ ^]^ (Mg[B(hfip)_4_]_2_/DME, cf. Figure 1) after initial cycling. Galvanostatic discharge–charge curves (1–200 cycles) are shown in Figure S1, Supporting Information, demonstrating that the active material remains reversibly electroactive and shows good cycling stability. Using elemental (EDX) mapping, we investigated the electrolyte distribution within the active material as well as the depletion of Cu from the active CuDEPP particles after initial voltammetric cycling. Further, we identified the electrochemical potential at which the Cu leaching starts by determining the Cu amount in the electrolyte at distinct potentials using total reflection X‐ray fluorescence spectrometry (TXRF). In addition, the formation of an inorganic salt‐rich interphase on the electrode was identified by laterally resolved time‐of‐flight secondary ion mass spectrometry (ToF‐SIMS).

## 2 Results and Discussion

### 2.1 Ex Situ TXRF Analysis of the Electrolyte

**Figure 2:**
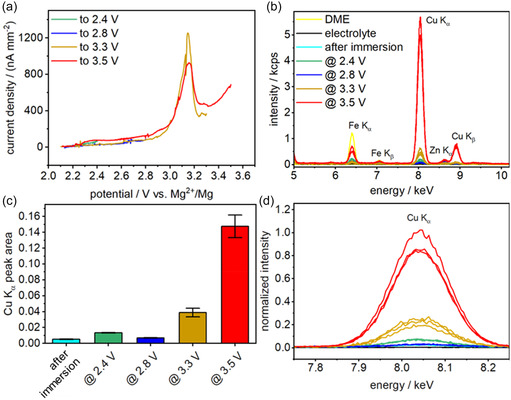
a) Linear sweep voltammograms of the CuDEPP electrodes from OCP to different potentials (2.4 V – green, 2.8 V – blue, 3.3 V – brown, and 3.5 V – red) in 0.3 m Mg[B(hfip)_4_]_2_/DME using a scan rate of 0.2 mV s^−1^. b) TXRF raw spectra of the electrolyte extracted for the Cu K_α_ and Cu K_β_ signals at the end potential given in (a); energy range from 5 to 10 keV. Fe and Zn are typical contaminants in reagents and materials. c) Bar chart of the peak areas of the normalized Cu K_α_ peaks shown in d). Bars represent the mean value of three replicates and error bars represent ± one standard deviation.

In a first step, to verify the leaching of Cu into the electrolyte and to determine the potential range at which this process occurs, CuDEPP composite electrodes were cycled using linear sweep voltammetry (LSV) (see **Figure** **2**a) up to distinct potentials versus Mg^2+^/Mg. The electrolyte was then extracted and investigated ex situ by TXRF (Figure 2b–d). The LSV data revealed a distinct electrochemical process evident at 3.1 V versus Mg^2+^/Mg, which was so far attributed to side reactions of the cathode with the electrolyte indicating the chemical conditioning that leads to a stabilized cathode behavior.^[^ ^35^ ^]^ Therefore, we have chosen the following potentials: 2.4 V (close to the open circuit potential (OCP)), 2.8 V (close to the distinct peak), 3.3 V (after the peak), and 3.5 V versus Mg^2+^/Mg.

Further, the solvent (DME), the freshly prepared electrolyte and the electrolyte after immersion of a CuDEPP electrode (without applied potential) for 140 min, which exceeds the time required for the LSV experiment (3.5 V vs. Mg^2+^/Mg), were also analyzed for reference. DME and the prepared electrolyte solution did not show any significant contamination of Cu. This is apparent from the TXRF analysis shown in Figure 2b,d (yellow and black, and in Figure S2, Supporting Information, showing the TXRF analysis energy range from 0 to 20 keV). Typical trace elements which were found during the TXRF analysis included iron (Fe) and zinc (Zn). These elements are ubiquitous contaminants in many reagents and materials, but the content of Cu was negligible. The electrolyte solution after immersion of the CuDEPP composite electrode (teal), as well as the samples extracted at 2.4 V (green) and 2.8 V (blue) versus Mg^2+^/Mg contained comparably low amounts of Cu. The apparent increase in Cu signal might indicate a minor dissolution of the active material in the electrolyte. However, a difference compared to the freshly prepared electrolyte is hardly visible for the immersed sample (cf. teal and black in Figure 2d). The increase of the Cu signal was ≈161% and 35% for the 2.4 and 2.8 V samples compared to the immersed one. Samples collected after cycling up to 3.3 V (brown) versus Mg^2+^/Mg, i.e., beyond the peak potential visible in Figure 2a, showed a significant increase in Cu content of about 659% and for the sample collected at 3.5 V (red) versus Mg^2+^/Mg, an increase of about 2789% compared to the immersed sample. This clearly points to an electrochemically driven mechanism responsible for the leaching of Cu into the electrolyte. Additionally, this may cause a change of the coordination environment resulting in a structural rearrangement of the active material, as Mg‐based porphyrins typically accommodate two axial ligands, whereas Cu‐based porphyrins have only one.^[^ ^38^ ^]^ In contrast, the stability of the porphyrins is opposing to this driving force since Cu‐based porphyrins are generally considered to be more stable than their Mg‐based counterparts.^[^ ^39^ ^]^ Therefore, this corroborates the assumption that an external driving force is required to supplant the Cu atom in the porphyrin structure.

### 2.2 SEM/EDX Analysis of Embedded Electrodes

To investigate the extent of Cu depletion in the active material in the electrodes during cycling, SEM/EDX analysis of embedded and stained electrode samples was performed. Electrode samples were vapor stained with osmium tetroxide (OsO_4_) and subsequently embedded in a silicone resin and polished to obtain cross‐sections as previously described.^[^ ^24^ ^]^ Embedding and polishing of the electrodes fill the pore space and removes topographical features and thereby minimizes artefacts, e.g., shine‐through artefacts and shadowing, while staining allows visualization and tracking of putative chemical changes in the active material.^[^ ^24^ ^]^

In a pristine sample, CuDEPP particles could be identified by their Cu content in the EDX maps, whereas no Mg was present since the electrode was not exposed to the Mg‐containing electrolyte (cf. Figures S3 and S4, Supporting Information). The observed osmium (Os) signal of the pristine sample was due to the presence of styrene butadiene rubber (SBR), whose carbon–carbon double bonds were stained by OsO_4_.^[^ ^40^ ^]^ However, Os was not present within the active CuDEPP material given the conjugated double bonds and the alkynyl moiety, which were not stained, as OsO4 reacts only with aliphatic (nonconjugated) double bonds to osmate esters, which has been previously reported^[^ ^24^ ^]^ (see also EDX measurements of individual CuDEPP particles shown in Figure S4, Supporting Information). Additionally, the spectra in Figure S4, Supporting Information, show that the active CuDEPP material was impregnated with the silicone resin and that Os was present in the cycled CuDEPP particles. Furthermore, the fluorine content in the cycled samples suggests the penetration of either the electrolyte anion [B(hfip)_4_]^−^, which is the only initial fluorine‐containing species, or its degradation products into the active material.

**Figure 3:**
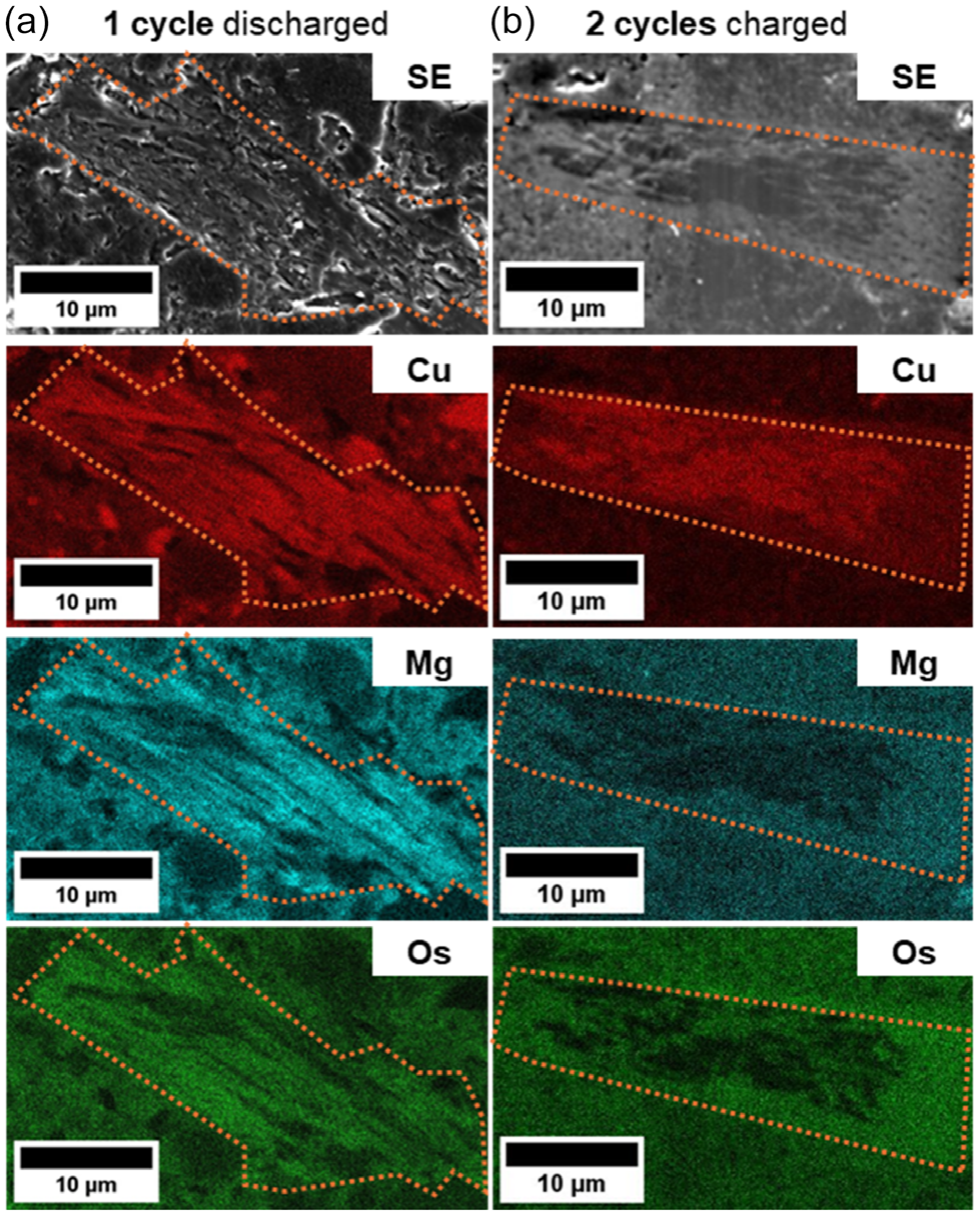
SE images (grayscale images) and elemental mappings showing the Cu (red), Os (green), and Mg (teal) distribution of the stained, embedded, and polished CuDEPP composite electrodes; a) after one cycle in the discharged state and b) after two cycles in the charged state. Orange dashed lines outline CuDEPP particles for clarity.

In the discharged state after one cycle, the electrode showed an enrichment of Mg within the CuDEPP particles, whereas the particles appear somewhat Mg‐depleted in the charged state after two cycles (cf. **Figure** **3**a,b). This trend coincides with the charge–discharge mechanism illustrated in Figure 1. However, the trend of Cu distribution appears to be the opposite. The edge areas of the particles were Cu‐depleted, whereas Mg remained within the particle (cf. Figure 3b). We hypothesize a transmetalation process by which the Cu center of the porphyrin is reduced and replaced by Mg^2+^‐ion from the electrolyte. This would explain the accumulation of Mg and depletion of Cu in the discharged state. As the inner core area of the particles still appears to be Cu‐rich, the electrochemical process of the initial cycles does not seem to be homogeneous throughout the particle. This is probably related to the penetration/insertion depth of the electrolyte into the active material. In addition, as this is a potential‐driven process and the active material is nonconductive, there is a potential gradient within the particle, which means that the insertion is likely limited by the particle sizes. The enrichment of Os within the particle is in agreement with a previous study revealing a self‐conditioning step of the CuDEPP during initial cycling.^[^ ^24^ ^]^

Since the stained pristine sample does not show a distinct presence of Os in the CuDEPP particles (cf. Figure S4, Supporting Information), there has to be a chemical change occurring during initial cycling of the CuDEPP composite electrodes allowing the staining of the active material. This change might be due to either an electrochemically induced polymerization involving the formation of double bonds, as has already been shown for monovalent battery chemistries using CuDEPP,^[^ ^24^ ^]^ or to a change or breakdown of the porphyrin structure. Further, Os staining within the CuDEPP particles appears only where Mg, i.e., the electrolyte, is already present. This might also be a result of electrolyte decomposition like a defluorination^[^ ^37^ ^]^ which hypothetically might also result in the formation of stainable double bonds.

### 2.3 Post‐Mortem Raman Spectroscopy of the Electrodes

In addition to the chemical changes that enable staining, the de‐ and insertion of the cation and anion into the particle during the initial cycles induce structural changes, which might also lead to a distortion or even to a partial breakdown of the porphyrin structure. To investigate these structural changes after cycling the electrode, Raman spectroscopy as well as ToF‐ and Orbi‐SIMS analysis were performed postmortem.

**Figure 4:**
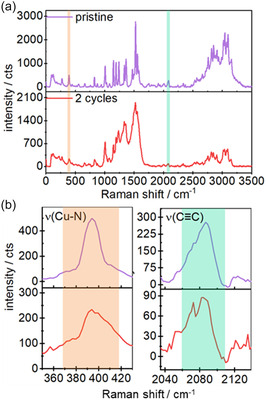
a) Raman spectra of an active CuDEPP particle of a pristine electrode (purple) and after two cycles in 0.3 m Mg[B(hfip)_4_]_2_/DME (red). b) Zoomed spectral range of the *ν*(C≡C) vibrational band (green area) and the *ν*(Cu–N) vibrational band (orange area). Presented Raman spectra are background corrected to account for fluorescence effects.

Since the depletion of Cu was most prominent in the sample already after two cycles in the charged state, the following Raman and ToF‐/Orbi‐SIMS analyses focused on these samples. The Raman spectrum of a pristine CuDEPP composite electrode (**Figure** **4**a, purple) shows characteristic narrow bands of the Cu–N stretching vibration at 391 cm^−1^ (marked in orange), the C≡C stretching vibration of the ethynyl moiety at 2086 cm^−1^ (marked in green), the stretching vibration of pyrrole‐substructure at 1335, 1365, and 1474 cm^−1^, the stretching vibration of the methine‐pyrrole bridging bond at 1526 and 1558 cm^−1^ as well as the stretching vibration between the phenyl substituent and the pyrrole carbon of the porphyrin structure at 1240 cm^−1^, which is in accordance with previously reported Raman studies of the CuDEPP.^[^ ^24^, ^41^ ^]^ After cycling (Figure 4, red), a general broadening of the bands indicates changes in the bond lengths within the material, most likely due to ion insertion from the electrolyte into the porphyrin structure, causing a distortion of the original structure.^[^ ^33^ ^]^ However, a marginal breakdown of the structure cannot be fully excluded and putative new bands, e.g., from stainable double bonds at around 1650 cm^−1^, are then most likely superimposed due to the band broadening (Figure 4a). Despite this, all main vibrational features of the pristine CuDEPP are still visible and distinguishable in the spectrum of the cycled sample. This indicates the preservation of the overall porphyrin structure during cycling. Interestingly, the vibrational band of the C≡C bond remained also intact after initial cycling (Figure 4b). This is in contrast to previous studies of monovalent ion batteries with CuDEPP as the active cathode material, in which the C≡C bond vibrational band was significantly reduced or disappeared completely after the initial cycling.^[^ ^23^, ^24^, ^33^, ^34^ ^]^ Those studies proposed that the active material undergoes an electrochemically induced polymerization *via* the ethynyl moiety. Therefore, it is assumed that the initial self‐conditioning in the case of the multivalent Mg^2+^‐ion does not include such an electrochemical polymerization step of the ethynyl moiety. Mg^2+^ in comparison to monovalent cations strongly coordinates to the alkyne's π‐system due to the high charge density and strong Lewis acidity. This strong coordination leads to a stabilization of these functional groups, reducing their reactivity, which may suppress alkyne polymerization processes and therefore might be the cause for the difference in behavior compared to monovalent cations.^[^ ^42^ ^]^ Additionally, the preservation of the triple‐bond discounts the formation of stainable double bonds. This implies that the accumulation of Os within the particle must be ascribed to a different process occurring during initial cycling.

One possible hypothesis is that the marginal breakdown of the structure is not distinguishable in the presented Raman spectra. This may due to band broadening and/or electrolyte degradation, resulting in stainable compounds, as discussed above. Regarding a possible transmetalation process, a shoulder becomes visible after cycling at the *ν*(Cu—N) vibrational band at 391 cm^−1^. This could be due to a distortion of the Cu—N bond or the convolution of an additional vibration band, e.g., a putative *ν*(Mg—N) band resulting from the substitution of the central Cu atom with a Mg atom.

### 2.4 ToF‐ and Orbi‐SIMS Analysis

To gain better insight into the composition and spatial distribution of the different compounds after cycling and to further elucidate degradation or transmetalation, a CuDEPP composite electrode was further investigated after two cycles (charged state) using ToF‐SIMS for high‐resolution ion images and Orbi‐SIMS for high‐resolution mass spectra. The electrode was embedded as described above (without staining) and cut into thin slices using a microtome (see Figure S5, Supporting Information) to obtain a flat surface in order to minimize topography‐related artefacts during the measurement. High‐resolution mass spectra and ion images were recorded in positive and negative polarity, respectively. The workflow was as follows: First, high‐resolution ion images were acquired using ToF‐SIMS in the so‐called delayed extraction mode. In short, this measurement mode enables high lateral resolution images combined with improved mass resolution.^[^ ^43^, ^44^ ^]^ Subsequently, multivariate statistical analysis in form of multivariate curve resolution (MCR) analysis of the ToF‐SIMS images was performed and suitable mass signals were selected based on the resulting factors. The mass signals in the recorded mass spectra were then identified and the signal assignments confirmed using the high‐resolution mass spectra of the Orbi‐SIMS measurements.

**Figure 5:**
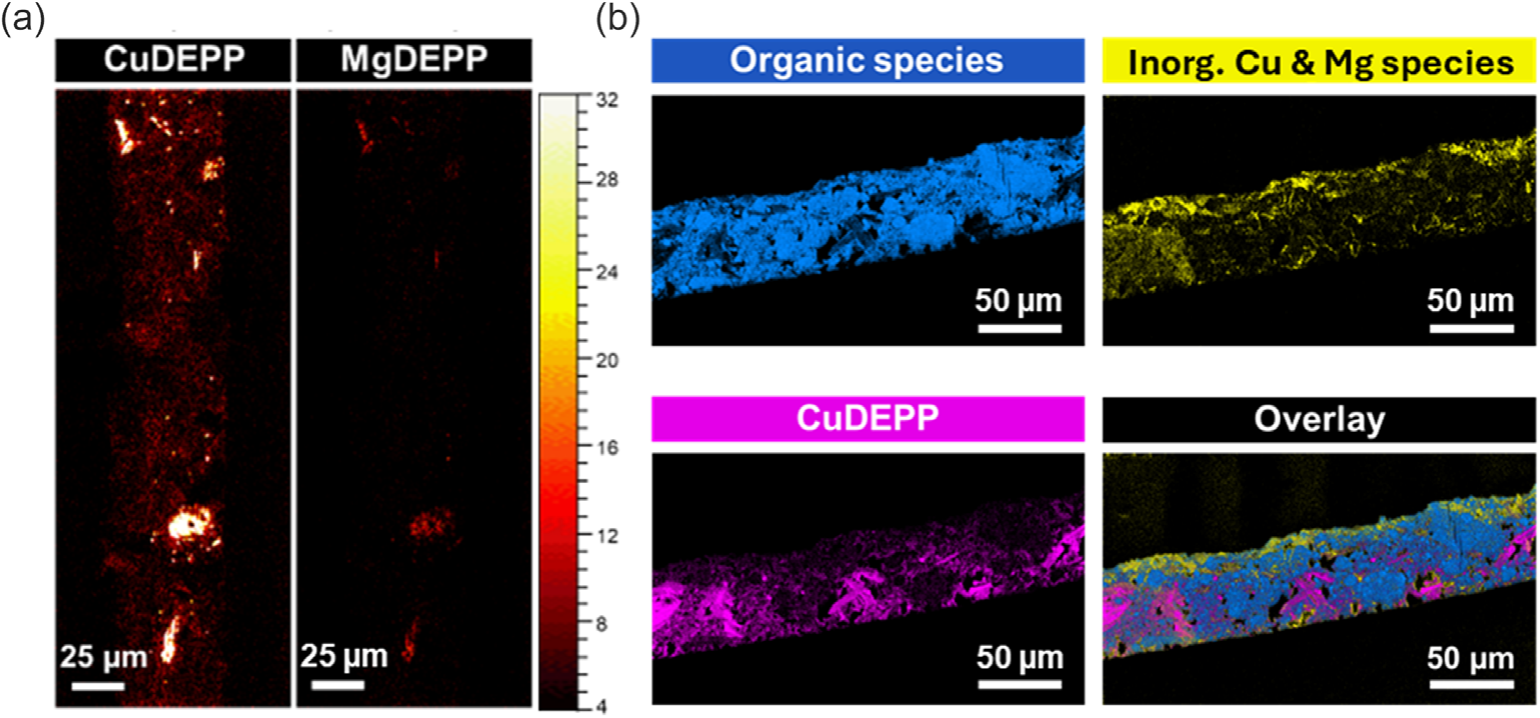
a) ToF‐SIMS ion images of a cycled CuDEPP composite electrode (two cycles, charged state) show lateral distribution of mass signals assigned to CuDEPP (CuC_x_N_y_, CuC_x_H_y_N_z_, and CuC_x_N_y_O_z_) and MgDEPP (MgC_x_N_y_ and MgC_x_H_y_N_z_). Measurement was performed in the delayed extraction mode, positive polarity, with 30 keV Bi_3_^+^ as primary ion species. b) Results of the performed MCR analysis of ToF‐SIMS images show organic components in blue (C_x_, C_x_H_y_, C_x_H_y_O_z_, C_x_H_y_N_z_, and C_n_H_x_N_y_O_z_), inorganic Cu and Mg species in yellow (Cu_x_Cl_y_, Cu_x_O_y_F_z_, Mg_x_Cu_y_Cl_z_, Mg_x_F_y_, Mg_x_Cl_y_, and Mg_x_F_y_Cl_z_), and CuDEPP in purple (CuC_x_N_y_, CuC_x_H_y_N_z_, and CuC_x_N_y_O_z_). Measurement was performed in delayed extraction mode, negative polarity, with 30 keV Bi_3_^+^ as primary ion species. The upper side was exposed to the electrolyte and the current collector facing side is the bottom side.

The ToF‐SIMS ion images obtained in positive ion mode show organic Cu fragments, i.e., CuC_x_N_y_, CuC_x_H_y_N_z_, and CuC_x_N_y_O_z_, occurring throughout the cycled composite electrode (**Figure** **5**a, left side; Figure S6, Supporting Information). This is consistent with the EDX analysis for Cu of the cycled electrode shown in Figure 3b. However, several agglomerates of high intensity were present that are ascribed to CuDEPP particles. Interestingly, organic Mg fragments, MgC_x_N_y_ and MgC_x_H_y_N_z_ (Figure 5a, right side), were mainly present in those regions within the active material, whereas other Mg species, i.e., Mg oxides or fluorides, were also detectable throughout the cycled electrode (Figure S7c,d, Supporting Information). This may be related to that either Mg is entrapped, and the charging of the material does not fully remove inserted Mg^2+^‐ions or that the Mg^2+^‐ion supplanted the central Cu atom of the porphyrin by a transmetalation step from CuDEPP to MgDEPP.

According to the MCR analysis of ToF‐SIMS images obtained in negative ion mode, different fragment types are clustered into different organic species (blue), inorganic Cu and Mg species (Cu_x_Cl_y_, Cu_x_O_y_F_z_, Mg_x_Cu_y_Cl_z_, Mg_x_F_y_, Mg_x_Cl_y_, and Mg_x_F_y_Cl_z_, marked in yellow) and CuDEPP (purple) species (Figure 5b). An accumulation of inorganic Cu and Mg species at the electrolyte‐facing side of the electrode becomes evident. The fluorinated Mg species strongly indicate the decomposition of the electrolyte. However, the presence of fluorinated Cu species is an indication that an interphase is formed with contribution from the active material, e.g., due to decomposition of the CuDEPP, which might also enable the staining with OsO_4_. Alternatively, the solvated Cu species may react with the electrolyte (cf. also Figure S6, Supporting Information, for zoomed depictions and additional overlays of the analyzed electrode). The presence of chloride species within this layer can either be attributed to trace contaminants in the electrolyte from synthesis or from the resins used for embedding. These inorganic species appear to be also present directly adjacent to active material (CuDEPP) inside the bulk of the electrode. As the CuDEPP molecule is fragmented by the ion beam, no information on possible polymerization steps during the initial cycles can be obtained from the ToF‐SIMS analysis.

## 3 Conclusion

Here, we could demonstrate that the active CuDEPP material undergoes chemical and structural changes during the initial cycles in magnesium tetrakis(hexafluoro‐isopropyloxy)borate in 1,2‐dimethoxyethane electrolyte. Combination of voltammetric cycling and TXRF analysis reveals significant Cu leaching from the CuDEPP beyond 3.1 V versus Mg^2+^/Mg, which is clearly associated with the electrochemical driving force. ToF‐ and Orbi‐SIMS analyses identify the formation of an inorganic halogen‐rich salt interphase on the electrolyte‐facing side of the CuDEPP composite electrode, indicating decomposition of the electrolyte in the initial cycles. In contrast to previous studies involving monovalent ion batteries, no clear evidence for polymerization of ethynyl moieties could be found. The recorded Raman spectra show only minute changes in the ethynyl band as well as all main bands of the original porphyrin structure, which indicates an overall retention of the original porphyrin framework, or the extent of degradation is too small and putative new bands are superimposed by band broadening. Further, Os staining experiments confirm that a chemical change occurs during the initial cycling since Os staining of the CuDEPP particles is absent in the particles of the pristine sample but clearly present in the cycled samples implying either a breakdown of the material or a new stainable species due to the electrolyte decomposition. These findings clearly demonstrate the differences of the same CuDEPP structure as active cathode material in mono‐ and divalent battery chemistries. Our data suggest a transmetalation, associated with Cu leaching. The combination of complementary instrumental techniques allowed us to gain insight into the structural and chemical changes during the first cycles. In future studies, the impact of the reported interphase formation will be studied.

## 4 Experimental Section

4.1

4.1.1

##### Fabrication and Cycling of CuDEPP Composite Electrodes

The synthesis of the active CuDEPP material is described elsewhere.^[^ ^23^ ^]^ The active material was mixed with graphene nanoplatelets with a surface area of 500 m^2^ g^−1^ (Sigma Aldrich, USA) and Super C65 (Imerys S.A., France) as conductive agents and sodium carboxymethylcellulose (Sunrose MAC 500, Nippon Paper Group, Japan) and SBR (VOLTABOND 029, Trinseo, USA) as binder in a mass relation of 59.6:23.2:3.0:2.3:1.9, respectively. The electrodes were processed from an aqueous slurry by casting on a carbon coated aluminum foil current collector, which was punched into discs (11.8 mm diameter) and then dried overnight at 80 °C. The mass loading of the electrodes was about 1.2 mg cm^−2^. The 0.3 m Mg[B(hfip)_4_]_2_/DME electrolyte was synthesized according to a previous study.^[^ ^45^ ^]^

For EDX analysis, as well as ToF‐ and Orbi‐SIMS studies, CR2032 coin‐type cells (Hohsen Corp., Japan) consisting of the CuDEPP composite cathode, a polypropylene film separator (PP, Celgard 2400), and a polished Mg anode were used for the cycling experiments. Galvanostatic cycling was performed using a VMP3 battery tester (Biologic, France) at a current density of 200 mA g^−1^.

For the TXRF studies of the electrolyte, pristine CuDEPP composite electrodes were cycled from their respective OCP to upper potential values of 2.4, 2.8, 3.3, and 3.5 V versus Mg^2+^/Mg, respectively. A three‐electrode set‐up (working electrode: CuDEPP composite electrode; counter electrode: Mg wire; reference electrode: Mg wire (both 1 mm diameter, Goodfellow GmbH, Germany) was used in a volume of 700 μL of 0.3 m Mg[B(hfip)_4_]_2_/DME) with a scan rate of 0.2 mV s^−1^ using a PGSTAT302N and the software Nova 2.1.4 (both Metrohm Autolab B.V., Utrecht, The Netherlands).

##### Staining, Embedding and Microtomy

For SEM and EDX analysis, the CuDEPP composite electrodes were embedded and stained: Small portions of the CuDEPP composite electrodes were cut and placed overnight in a sealed container with a droplet of 4 wt% aqueous OsO_4_ solution. Highly volatile OsO_4_ sublimates and reacts with aliphatic double bonds present in the sample accumulating Os.^[^ ^46^, ^47^ ^]^ To minimize artefacts during elemental mapping and SE imaging and to enhance contrast between carbon‐based electrode components and pore space, the samples were embedded using a two‐step embedding procedure. First, the sample was infiltrated with silicone rubber (ELASTOSIL RT 601 A/B, Wacker Chemie AG, Germany) aided by the application of two vacuum steps (240 mbar, 5 and 30 min) and hardened for 24 h at room temperature. Afterward, excess silicone rubber was removed by cutting and the samples were additionally embedded in epoxy resin (EpoFix, Struers GmbH, Germany) using the same embedding procedure with subsequent hardening for 24 h. Using SiC abrasive paper and diamond particle suspensions (monocrystalline 3 and 1 μm, Leco Corporation, USA) on Nylon paper (Leco Corporation, USA), the samples were polished to expose the embedded electrode sample and to obtain a flat surface. Finally, a ≈10 nm‐thick platinum layer was sputtered onto the samples to minimize charging effects during analysis.

For Raman and ToF‐SIMS analysis, the samples were embedded without OsO_4_ staining directly within epoxy resin and cut into ≈700–800 nm‐thick cross‐sections with a microtome (EM UC7, Leica Microsystems GmbH, Germany) and were placed on a silicon substrate (cf. Figure S5, Supporting Information).

##### Scanning Electron Microscopy and Energy‐Dispersive X‐Ray Spectroscopy

SE imaging and EDX mapping of the embedded and stained electrode samples were performed with a Quanta FEG 3D (Thermo Fisher Scientific, USA) equipped with an EDAX Element EDS system using the Apex software V3 (both EDAX Inc., Germany). For the EDX elemental mappings, 1024 pixels × 800 pixels were recorded using a dwell time of 100 μs and 64 frames at 6 kV accelerating voltage and a spot current of 8 nA.

##### ToF‐ and Orbi‐SIMS Measurements

ToF‐ and Orbi‐SIMS analyses were performed on an M6 Hybrid SIMS (IONTOF GmbH, Münster, Germany), which is equipped with both a ToF and Q‐Exactive Orbitrap mass analyzer. Measurements were carried out in positive and negative ion mode, respectively.

For high‐resolution ToF‐SIMS images, the liquid ion metal gun (LMIG) was operated in the fast‐imaging mode at a beam current of 0.015 pA and delayed extraction mode as well as the topography mode of the analyzer were activated. Imaging was conducted with 30 keV Bi_3_^+^ primary ions at a cycle time of 110 μs with delay time of 0.1 μs. For charge compensation, a low‐energy electron flood gun was applied as well as argon gas regulation at 10^−7^ mbar. Samples were analyzed on areas of 300 μm × 300 μm with 2048 pixels × 2048 pixels, one shot per pixel per frame, one frame, 75 scans, and a total primary ion dose of roughly 10^14^ cm^−2^. A mass resolution m/Δm >3800 @ *m/z* 63 (Cu^+^) was obtained. Each SI image was normalized to the respective total ion image.

For high‐resolution mass spectra, the orbitrap mass analyzer was used. Prior to Orbi‐SIMS spectra measurements, orbitrap mass calibration was performed on a silver reference once within 24 h. As primary ion gun, the LMIG was used and operated with 20% long pulses and 700 μm aperture. A single scan was performed for each image with areas of 100 μm × 100 μm. Sample areas were rastered in random mode with pixel size of 2 μm. The analyzer (all‐purpose mode) was set to a maximum injection time of 500 ms, a cycle time of 100 μs, and a mass range of 50–750 *m*/*z*, corresponding to a mass resolution of m/Δm 240 000 @ *m/z* 200. Each SI image was normalized to the respective total ion image.

Data were analyzed using software package SurfaceLab 7.4 (IONTOF GmbH, Münster, Germany). MCR image analyses were conducted to identify mass signals correlated with organic, CuDEPP, and MgDEPP species.

##### Total Reflection X‐Ray Fluorescence Spectrometry

TXRF was performed using high‐efficiency module S2 Picofox (Bruker Nano GmbH, Berlin, Germany) equipped with molybdenum X‐ray tube. The samples were excited using a voltage of 50 kV and a current of 600 μA. Measurement live time was set to 1000 s. After appropriate dilution of the samples using ultrapure water, 10 μL of titanium (Ti) standard solution (100 mg L^−1^ in 2 wt% HNO_3_, Honeywell Fluka, Charlotte, North Carolina, USA) was added to 500 μL of the diluted solution as an internal standard. After homogenization using a Vortex mixer (60 s; 2500 rpm), 10 μL of each sample was applied to a precleaned and siliconized quartz glass sample carrier. For evaporation of the solvent, the sample carriers were dried on a heating plate at 60 °C until complete dryness. For each sample, three replicates were prepared (*n* = 3).

For relative comparison of the Cu content in the electrolyte samples the TXRF spectra were normalized to the maximal signal intensity of the added Ti standard at 4.5 keV. The area of the Cu K_α_ peak was then integrated using the software package OriginPro 2023b SR1 10.0.5.157 where the measurement of the electrolyte was used as baseline.

##### Raman Spectroscopy

Raman spectra were recorded using a confocal Raman microscope (alpha 300 R, WITec GmbH, Germany) equipped using a green laser with an excitation wavelength of 532 nm. Low laser intensities smaller than 0.5 mW were used to minimize potential damage to the sample. Integration times of 1 s were chosen and a rounded shape background subtraction was performed to remove fluorescence effects *via* the software Project FIVE 5.0.6.46 (WITec GmbH, Germany).

## Conflict of Interest

The authors declare no conflict of interest.

## Supporting information

Supplementary Material

## Data Availability

The data that support the findings of this study are openly available in [Zenodo] at [DOI: 10.5281/zenodo.15742414], reference number [15742414].

## References

[1] L. Trahey , F. R. Brushett , N. P. Balsara , G. Ceder , L. Cheng , Y.‐M. Chiang , N. T. Hahn , B. J. Ingram , S. D. Minteer , J. S. Moore , K. T. Mueller , L. F. Nazar , K. A. Persson , D. J. Siegel , K. Xu , K. R. Zavadil , V. Srinivasan , G. W. Crabtree , Energy storage emerging: A perspective from the Joint Center for Energy Storage Research, Proc. Natl. Acad. Sci. 2020, 117, 12550. 32513683 10.1073/pnas.1821672117 PMC7293617

[2] A. Hamdan , C. D. Daudu , A. Fabuyide , E. A. Etukudoh , S. Sonko , Next‐generation batteries and U.S. energy storage: A comprehensive review: Scrutinizing advancements in battery technology, their role in renewable energy, and grid stability, World J. Adv. Res. Rev. 2024, 21, 1984.

[3] H. Chen , M. Armand , G. Demailly , F. Dolhem , P. Poizot , J. M. Tarascon , From Biomass to a Renewable LiXC6O6 Organic Electrode for Sustainable Li-Ion Batteries, ChemSusChem 2008, 1, 348. 18605101 10.1002/cssc.200700161

[4] Z. Xu , H. Ye , H. Li , Y. Xu , C. Wang , J. Yin , H. Zhu , Enhanced Lithium Ion Storage Performance of Tannic Acid in LiTFSI Electrolyte, ACS Omega 2017, 2, 1273. 31457503 10.1021/acsomega.6b00504 PMC6641128

[5] J. J. Shea , C. Luo , Organic Electrode Materials for Metal Ion Batteries, ACS Appl. Mater. Interfaces 2020, 12, 5361. 31917538 10.1021/acsami.9b20384

[6] S. J. Visco , C. C. Mailhe , L. C. De Jonghe , M. B. Armand , A Novel Class of Organosulfur Electrodes for Energy Storage, J. Electrochem. Soc. 1989, 136, 661.

[7] H. Zhu , J. Yin , X. Zhao , C. Wang , X. Yang , Humic acid as promising organic anodes for lithium/sodium ion batteries, Chem. Commun. 2015, 51, 14708. 10.1039/c5cc04772b 26269107

[8] K. Amin , Q. Meng , A. Ahmad , M. Cheng , M. Zhang , L. Mao , K. Lu , Z. Wei , A Carbonyl Compound‐Based Flexible Cathode with Superior Rate Performance and Cyclic Stability for Flexible Lithium‐Ion Batteries, Adv. Mater. 2018, 30, 201703868. 10.1002/adma.201703868 29226388

[9] J. C. Carlberg , O. Inganäs , Poly(3,4‐ethylenedioxythiophene) as Electrode Material in Electrochemical Capacitors, J. Electrochem. Soc. 1997, 144, L61.

[10] J. Chen , J. Wang , C. Wang , C. O. Too , G. G. Wallace , Lithium–Polymer battery based on polybithiophene as cathode material, J. Power Sources 2006, 159, 708.

[11] D.‐Y. Wang , R. Liu , W. Guo , G. Li , Y. Fu , Recent advances of organometallic complexes for rechargeable batteries, Coord. Chem. Rev. 2021, 429, 213650.

[12] A. Vizintin , J. Bitenc , A. Kopač Lautar , J. Grdadolnik , A. Randon Vitanova , K. Pirnat , Redox Mechanisms in Li and Mg Batteries Containing Poly(phenanthrene quinone)/Graphene Cathodes using Operando ATR‐IR Spectroscopy, ChemSusChem 2020, 13, 2328. 32052586 10.1002/cssc.202000054 PMC7317575

[13] Y. Wang , Z. Liu , C. Wang , Y. Hu , H. Lin , W. Kong , J. Ma , Z. Jin , π‐Conjugated polyimide‐based organic cathodes with extremely‐long cycling life for rechargeable magnesium batteries, Energy Storage Mater. 2020, 26, 494.

[14] D. Tao , Y. Tang , H. Gui , Y. Cao , F. Xu , Imine‐Based Conjugated Polymers as High‐Performance Organic Cathodes for Rechargeable Magnesium Batteries: Insights from Electronic Structure Design, Batter Supercaps 2025, 2500226.

[15] Z. Zhao‐Karger , P. Gao , T. Ebert , S. Klyatskaya , Z. Chen , M. Ruben , M. Fichtner , New Organic Electrode Materials for Ultrafast Electrochemical Energy Storage, Adv. Mater. 2019, 31, 1806599. 10.1002/adma.201806599 30786067

[16] Y. Guo , W. Wang , K. Guo , X. Chen , M. Wang , Z. Huang , Y. Zhu , W. Song , S. Jiao , A bipolar‐redox tetraalkynylporphyrin macrocycle positive electrode with 12‐electrons‐transfer for high‐energy aluminum‐organic batteries, Nat. Commun. 2025, 16, 2794. 40118862 10.1038/s41467-025-58126-5 PMC11928495

[17] M. Xie , J. Liu , L. Dai , H. Peng , Y. Xie , Advances and prospects of porphyrin derivatives in the energy field, RSC Adv. 2023, 13, 24699. 37601600 10.1039/d3ra04345b PMC10436694

[18] J. Min Park , J. H. Lee , W.‐D. Jang , Applications of porphyrins in emerging energy conversion technologies, Coord. Chem. Rev. 2020, 407, 213157.

[19] H. Wang , Q. Wu , L. Cheng , L. Chen , M. Li , G. Zhu , Porphyrin‐ and phthalocyanine‐based systems for rechargeable batteries, Energy Storage Mater. 2022, 52, 495.

[20] Y. Sun , F. He , X. Huang , B. Ren , J. Peng , D. Chen , X. Hu , X. Sun , P. Gao , Ethynyl functionalized porphyrin complex as a new cathode for organic alkali metal batteries with excellent cycling stability, Chem. Eng. J. 2023, 451, 138734.

[21] S. Chowdhury , S. Jana , S. P. K. Panguluri , W. Wenzel , S. Klayatskaya , M. Ruben , Ferrocene Appended Porphyrin‐Based Bipolar Electrode Material for High‐Performance Energy Storage, ChemSusChem 2024, 17, e202301903. 38266158 10.1002/cssc.202301903

[22] X. Huang , Y. Zhou , Y. Zeng , X. Chen , F. He , T. Wang , D. Lan , W. Liu , S. Tan , P. Gao , Electron‐donating/withdrawing groups functionalized porphyrin complex as high performance organic lithium batteries, Chem. Eng. J. 2023, 470, 144248.

[23] P. Gao , Z. Chen , Z. Zhao‐Karger , J. E. Mueller , C. Jung , S. Klyatskaya , T. Diemant , O. Fuhr , T. Jacob , R. J. Behm , M. Ruben , M. Fichtner , A Porphyrin Complex as a Self‐Conditioned Electrode Material for High‐Performance Energy Storage, Angew. Chem. Int. Ed. 2017, 56, 10341. 10.1002/anie.201702805 28627132

[24] T. Philipp , G. Neusser , E. Abouzari‐Lotf , S. Shakouri , F. D. H. Wilke , M. Fichtner , M. Ruben , M. Mundszinger , J. Biskupek , U. Kaiser , P. Scheitenberger , M. Lindén , C. Kranz , Visualization of structural changes and degradation of porphyrin‐based battery electrodes, J Power Sources 2022, 522, 231002.

[25] T. Smok , Y. Hu , S. Jana , F. Pammer , M. Fichtner , Exploring the chemical and structural change of copper porphyrins upon charging by means of synchrotron X‐ray absorption spectroscopy, Energy Adv. 2024, 3, 2348.

[26] C. K. Jung , D. Stottmeister , T. Jacob , Properties and Structural Arrangements of the Electrode Material CuDEPP during Energy Storage, Energy Technol. 2020, 8, 2000388.

[27] S. Shakouri , E. Abouzari‐Lotf , J. Chen , T. Diemant , S. Klyatskaya , F. D. Pammer , A. Mizuno , M. Fichtner , M. Ruben , Molecular Engineering of Metalloporphyrins for High‐Performance Energy Storage: Central Metal Matters, ChemSusChem 2023, 16, e202202090. 36445802 10.1002/cssc.202202090 PMC10107660

[28] Y. Zeng , J. Zhou , J. Zhang , F. He , Y. Su , P. Wang , S. Tan , P. Gao , Ultra‐long cycle life organic‐sodium batteries enabled by thiophene‐based porphyrin in‐situ electropolymerization, Chem. Eng. J. 2023, 453, 139951.

[29] J. Zhang , C. Ye , Y. Liao , C. Sun , Y. Zeng , J. Xiao , Z. Chen , W. Liu , X. Yang , P. Gao , Thiophene‐functionalized porphyrin complexes as high performance electrodes for sodium ion batteries, Mater. Futures 2023, 2, 035101.

[30] T. Smok , S. Shakouri , E. Abouzari‐Lotf , F. Pammer , T. Diemant , S. Jana , A. Roy , Y. Xiu , S. Klyatskaya , M. Ruben , Z. Zhao‐Karger , M. Fichtner , A π‐Conjugated Porphyrin Complex as Cathode Material Allows Fast and Stable Energy Storage in Calcium Batteries, Batter Supercaps 2023, 6, e202300308.

[31] X. Han , S. Li , W. Song , N. Chen , H. Chen , S. Huang , S. Jiao , Stable High‐Capacity Organic Aluminum–Porphyrin Batteries, Adv. Energy Mater. 2021, 11, 2101446.

[32] S. Chowdhury , N. Sabi , R. C. Rojano , N. Le Breton , A. K. Boudalis , S. Klayatskaya , S. Dsoke , M. Ruben , π‐Conjugated Metal Free Porphyrin as Organic Cathode for Aluminum Batteries, Batter Supercaps 2024, 7, e202300285.

[33] X. Chen , X. Feng , B. Ren , L. Jiang , H. Shu , X. Yang , Z. Chen , X. Sun , E. Liu , P. Gao , High Rate and Long Lifespan Sodium‐Organic Batteries Using Pseudocapacitive Porphyrin Complexes‐Based Cathode, Nanomicro Lett. 2021, 13, 71. 34138295 10.1007/s40820-021-00593-8 PMC8187698

[34] S. Lv , J. Yuan , Z. Chen , P. Gao , H. Shu , X. Yang , E. Liu , S. Tan , M. Ruben , Z. Zhao‐Karger , M. Fichtner , Copper Porphyrin as a Stable Cathode for High‐Performance Rechargeable Potassium Organic Batteries, ChemSusChem 2020, 13, 2286. 32187437 10.1002/cssc.202000425

[35] E. Abouzari‐Lotf , R. Azmi , Z. Li , S. Shakouri , Z. Chen , Z. Zhao‐Karger , S. Klyatskaya , J. Maibach , M. Ruben , M. Fichtner , A Self‐Conditioned Metalloporphyrin as a Highly Stable Cathode for Fast Rechargeable Magnesium Batteries, ChemSusChem 2021, 14, 1840. 33646642 10.1002/cssc.202100340 PMC8251709

[36] X. Feng , X. Chen , B. Ren , X. Wu , X. Huang , R. Ding , X. Sun , S. Tan , E. Liu , P. Gao , Stabilization of Organic Cathodes by a Temperature‐Induced Effect Enabling Higher Energy and Excellent Cyclability, ACS Appl. Mater. Interfaces 2021, 13, 7178. 33538571 10.1021/acsami.0c20525

[37] P. Jankowski , Z. Li , Z. Zhao‐Karger , T. Diemant , M. Fichtner , T. Vegge , J. M. G. Lastra , Development of Magnesium Borate Electrolytes: Explaining the Success of Mg[B(hfip)4]2 Salt, Energy Storage Mater. 2022, 45, 1133.

[38] J. R. Miller , G. D. Dorough , Pyridinate Complexes of Some Metallo‐derivatives of Tetraphenylporphine and Tetraphenylchlorin^1^ , J. Am. Chem. Soc. 1952, 74, 3977.

[39] M. Zerner , M. Gouterman , Porphyrins IV. Extended Hückel Calculations on Transition Metal Complexes, Theoret. Chim. Acta 1966, 4, 44.

[40] J.‐H. Lee , J. Kim , M. H. Jeong , K. H. Ahn , H. L. Lee , H. J. Youn , Visualization of styrene‐butadiene rubber (SBR) latex and large‐scale analysis of the microstructure of lithium‐ion battery (LIB) anodes, J. Power Sources 2023, 557, 232552.

[41] X.‐M. Lin , D.‐Y. Wu , P. Gao , Z. Chen , M. Ruben , M. Fichtner , Monitoring the Electrochemical Energy Storage Processes of an Organic Full Rechargeable Battery via Operando Raman Spectroscopy: A Mechanistic Study, Chem. Mater. 2019, 31, 3239.

[42] Y. Liang , U. K. Das , J. Luo , Y. Diskin‐Posner , L. Avram , D. Milstein , Magnesium Pincer Complexes and Their Applications in Catalytic Semihydrogenation of Alkynes and Hydrogenation of Alkenes: Evidence for Metal–Ligand Cooperation, J. Am. Chem. Soc. 2022, 144, 19115. 36194894 10.1021/jacs.2c08491 PMC9585592

[43] T. Lombardo , F. Walther , C. Kern , Y. Moryson , T. Weintraut , A. Henss , M. Rohnke , ToF‐SIMS in battery research: Advantages, limitations, and best practices, J. Vac. Sci. Technol., A 2023, 41, 053207.

[44] C. Kern , S. Kern , A. Henss , M. Rohnke , Secondary ion mass spectrometry for bone research, Biointerphases 2023, 18, 041203. 37489909 10.1116/6.0002820

[45] Z. Zhao‐Karger , R. Liu , W. Dai , Z. Li , T. Diemant , B. P. Vinayan , C. Bonatto Minella , X. Yu , A. Manthiram , R. J. Behm , M. Ruben , M. Fichtner , Toward Highly Reversible Magnesium‐Sulfur Batteries with Efficient and Practical Mg[B(hfip)_4_]_2_ Electrolyte, ACS Energy Lett. 2018, 3, 2005.

[46] M. A. Parker , D. Vesely , Contrast enhancement and polymer identification in the electron microscope by the formation and staining of unsaturated double bonds, Microsc. Res. Tech. 1993, 24, 333. 7685644 10.1002/jemt.1070240406

[47] G. H. Michler , Electron Microscopy of Polymers, Springer Berlin Heidelberg, Berlin, Heidelberg 2008.

