# Supplementary material for: Metalloporphyrin‐Based Cathode for Rechargeable Magnesium‐Ion Batteries: Copper Leaching and Interphase Formation
Source: ChemSusChem. 2025 Nov 11;19(7):e202501463. doi: 10.1002/cssc.202501463 (PMC13080235; doi:10.1002/cssc.202501463)
Supplement: Supplementary file 1 — Supplementary Material [file CSSC-19-e202501463-s001.pdf]

Supporting Information

Metalloporphyrin-based cathode for rechargeable magnesium-ion batteries: copper leaching and interphase formation

Tom Philipp[a], Riccarda Müller[a], Till Ortmann[b], Christine Kern[b], Marcus Rohnke[b], Simon Schauer[c], Mika Lindén[c], Ebrahim Abouzari-Lotf[d], Thomas Smok[d], Maximilian Fichtner[d], Shirin Shakouri[e], Mario Ruben[e,f,g], Kerstin Leopold[a], and Christine Kranz*[a]

[a] Tom Philipp, Riccarda Müller, Prof. Dr. Kerstin Leopold, Prof. Dr. Christine Kranz
Institute of Analytical and Bioanalytical Chemistry (IABC)
Ulm University
Albert-Einstein-Allee 11, 89081 Ulm, Germany
E-mail: christine.kranz@uni-ulm.de

[b] Dr. Till Ortmann, Christine Kern, Prof. Dr. Marcus Rohnke
Institute of Physical Chemistry and Center for Materials Research
Justus Liebig University Giessen
Heinrich-Buff-Ring 17, 35392 Giessen, Germany

[c] Simon Schauer, Prof. Dr. Mika Lindén
Institute of Inorganic Chemistry II
Ulm University
Albert-Einstein-Allee 11, 89081 Ulm, Germany

[d] Dr. Ebrahim Abouzari-Lotf, Thomas Smok, Prof. Dr. Maximilian Fichtner
Solid State Chemistry,
Helmholtz Institute Ulm (HIU)
Helmholtzstraße 11, 89081 Ulm, Germany

Institute of Nanotechnology (INT)
Karlsruhe Institute of Technology (KIT)
Hermann-von-Helmholtz-Platz 1, 76344 Eggenstein-Leopoldshafen, Germany

[e] Dr. Shirin Shakouri, Prof. Dr. Mario Ruben
Institute of Nanotechnology (INT)
Karlsruhe Institute of Technology (KIT)
Hermann-von-Helmholtz-Platz 1, 76344 Eggenstein-Leopoldshafen, Germany

[f] Prof. Dr. Mario Ruben
Institute of Quantum Materials and Technologies (IQMT)
Karlsruhe Institute of Technology (KIT)
Kaiserstraße 12, 76131 Karlsruhe, Germany

[g] Prof. Dr. Mario Ruben
Centre Européen de Sciences Quantiques (CESQ)
Institut de Science et d'Ingénierie Supramoléculaires (ISIS)
8 allée Gaspard Monge, BP 70028, 67083 Strasbourg Cedex, France

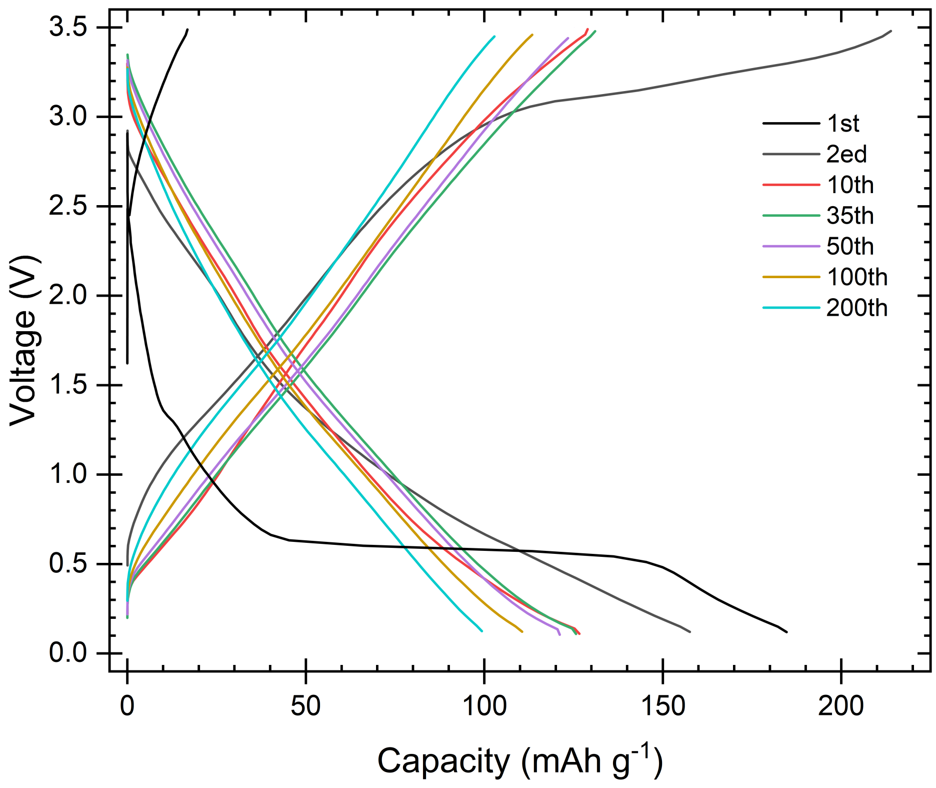

Figure S1: Selected galvanostatic discharge-charge profiles of the CuDEPP composite electrode in the potential range of 0.1–3.5 V vs. Mg^{2+}/Mg with Mg/Mg[B(hfip)$_4$]$_2$ electrolyte at 1 A g^{-1} and a cutoff voltage of 3.5 V.

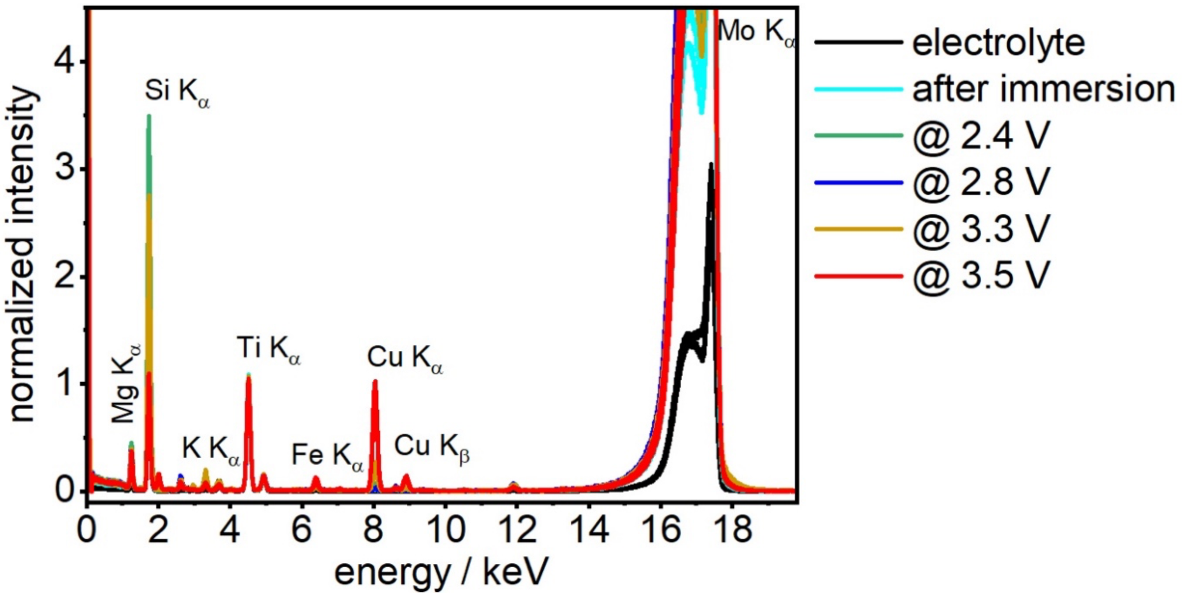

Figure S2: TXRF analysis of electrolyte samples extracted at the given end potentials including the electrolyte as prepared and after immersion of a CuDEPP composite electrode for 140 minutes. The intensities are normalized

to the Ti K_{α} signal. Cu originates from the active material, Mg from the electrolyte, Si from the sample holder, K, Fe and Zn are typical contaminants of reagents and materials, Ti was used as internal standard, Mo originates from the used excitation source.

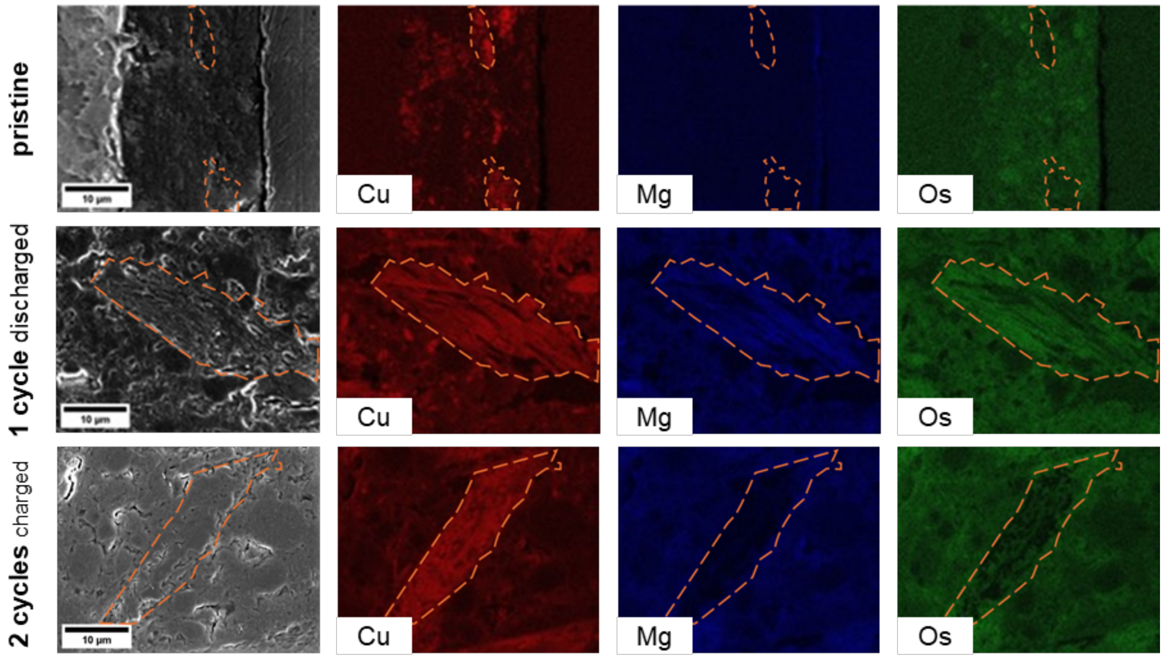

Figure S3: Elemental maps of Cu, N, Os and Mg of a stained and embedded CuDEPP electrodes: pristine, after one cycle (discharged state) and after two cycles (charged state).

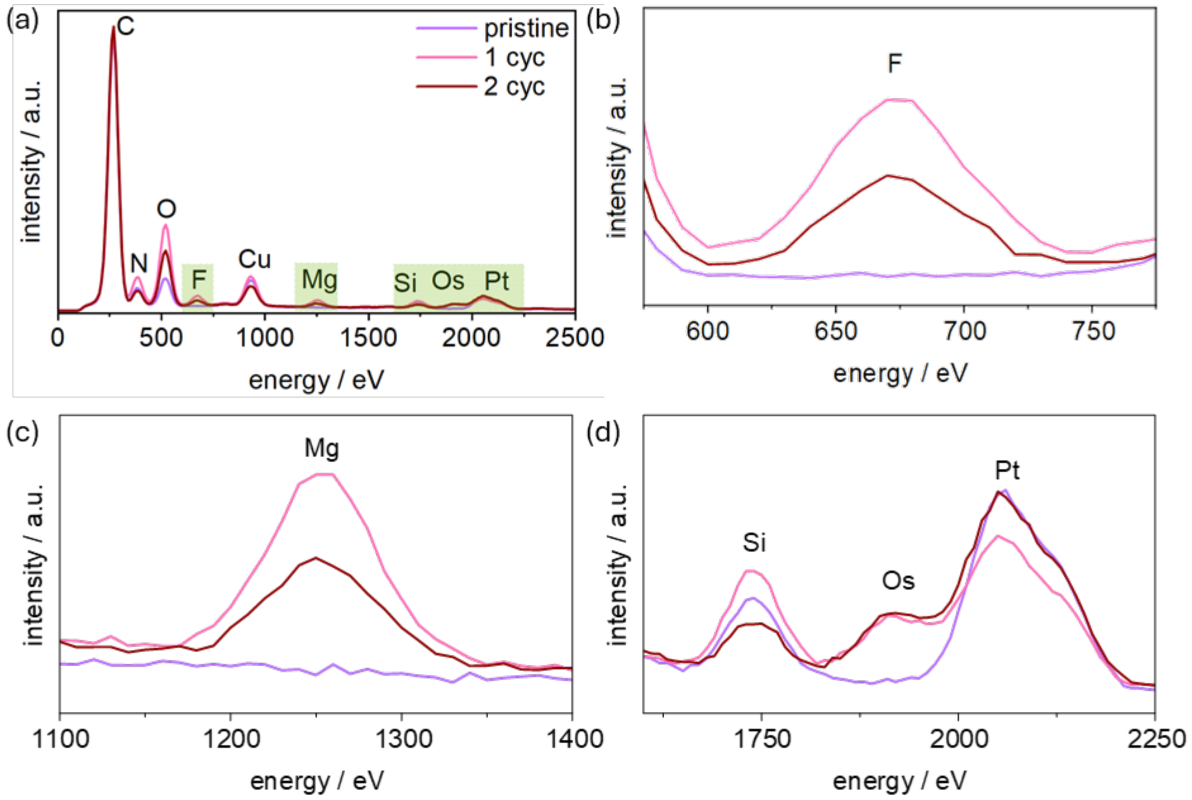

Figure S4: (a) EDX spectra recorded from a CuDEPP particle in an embedded pristine electrode (purple), after one cycle (pink) and after two cycles (dark red). (b-d) Zoomed-in EDX spectra of the green marked ranges in (a) for fluorine (F), magnesium (Mg), silicon (Si), osmium (Os) and platinum (Pt).

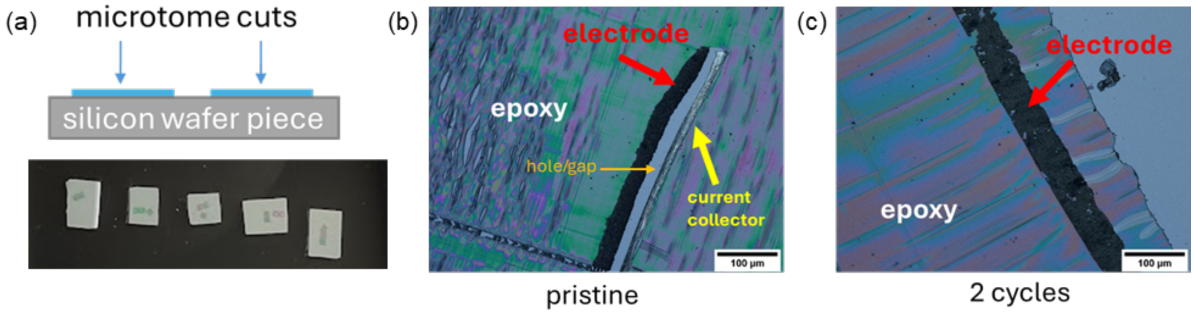

Figure S5: (a) Schematic (top) and photograph (bottom) of the microtome cut samples on a silicon wafer substrate for ToF-SIMS and Raman analysis. (b) Optical image of a cut pristine sample and (c) of a cycled sample (2 cycles). In the pristine sample the current collector is still present whereas the current collector separated during cell disassembly for the cycled samples.

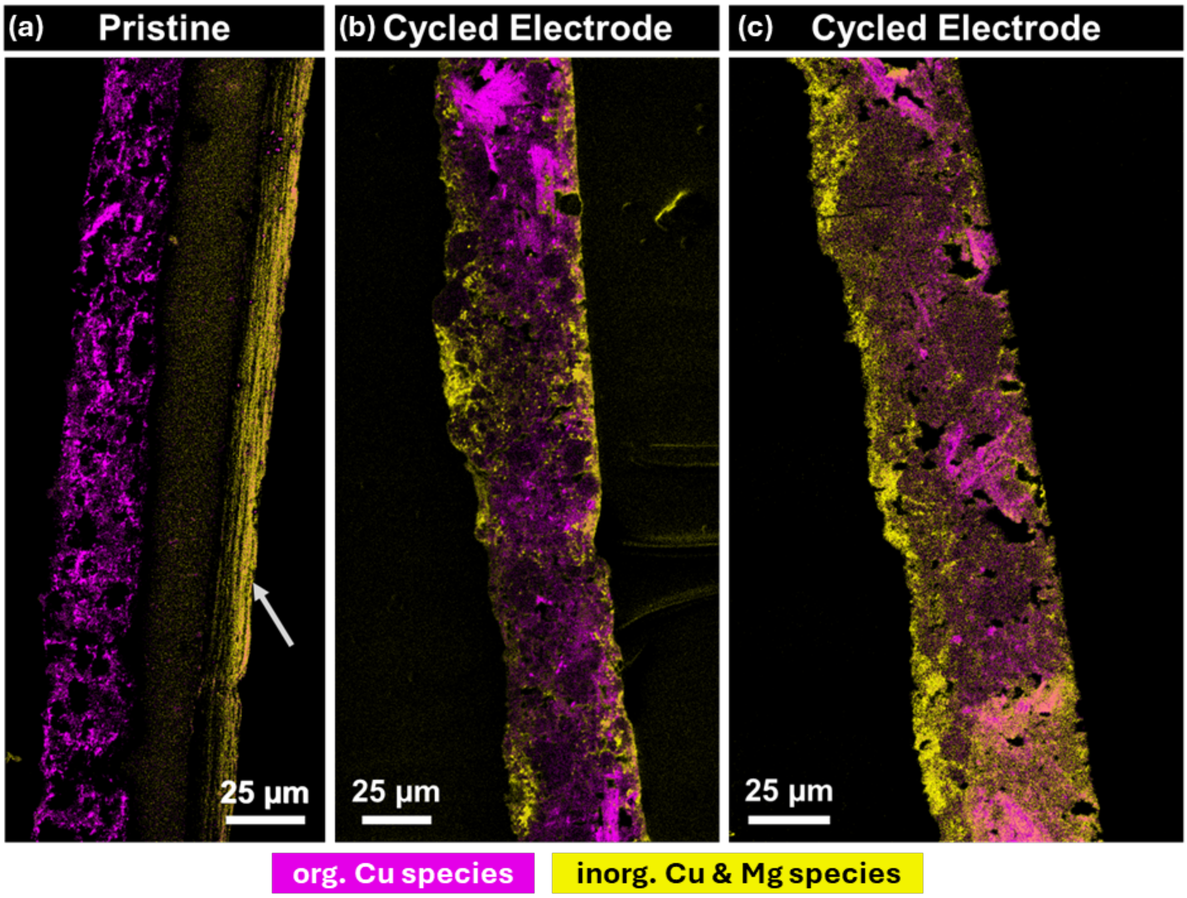

Figure S6: ToF-SIMS ion images of microtome cut CuDEPP composite electrodes: (a) pristine and (b and c) two areas of an electrode after two cycles. Delayed extraction mode, negative polarity, 30 keV Bi_3^+ as primary ion species. Organic copper species ($CuC_xN_y^-$, $CuC_xH_yN_z^-$, $CuC_xN_yO_z^-$) are depicted in purple, inorganic Mg and Cu species ($Cu_xCl_y^-$, $Cu_zO_yF_z^-$, $Mg_xCu_yCl_z^-$, $Mg_xF_y^-$, $Mg_xCl_y^-$, $Mg_xFl_yCl_z^-$) are depicted in yellow. Gray arrow indicates the current collector in the pristine sample, right side of the cycled electrodes was facing the current collector.

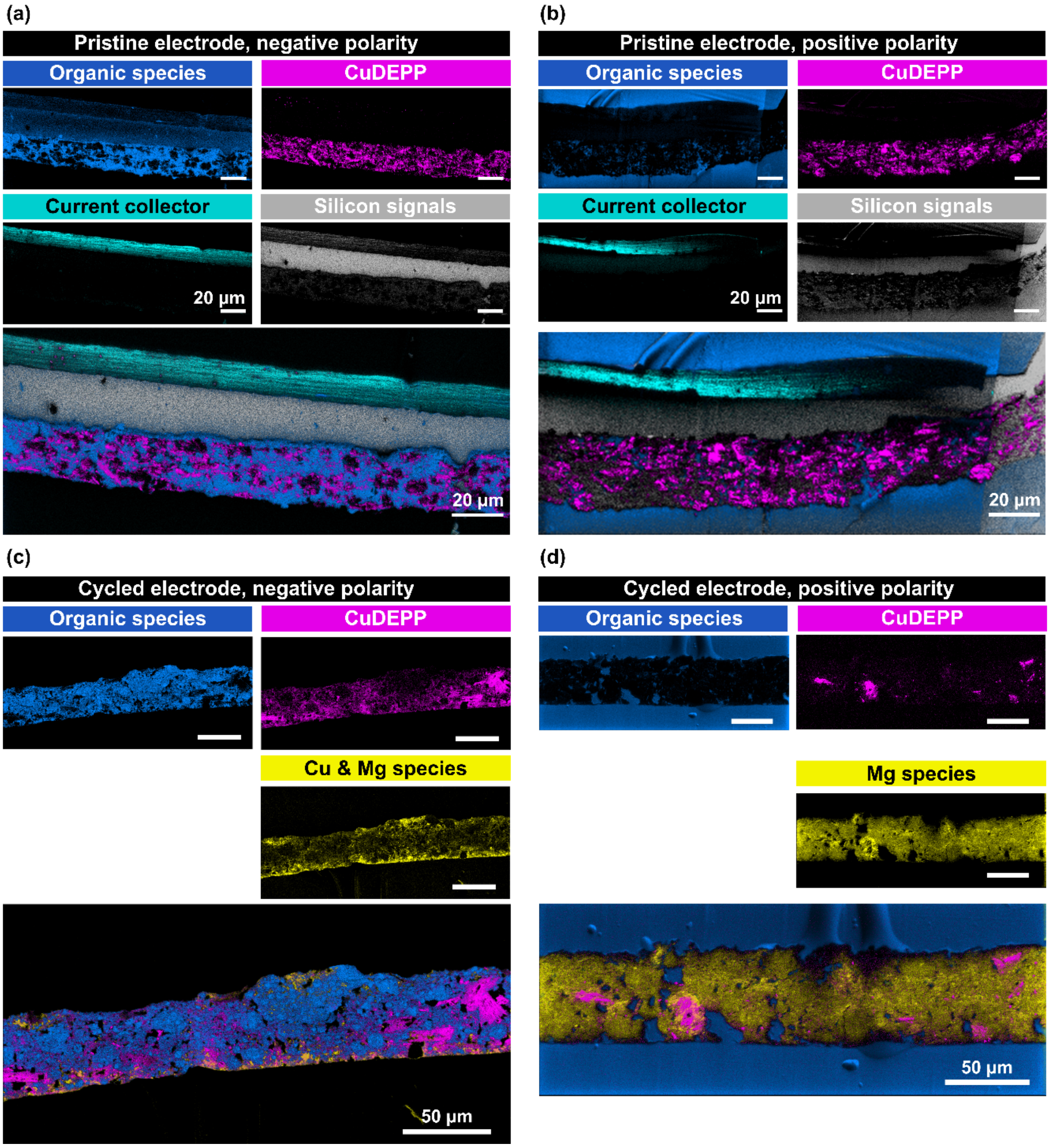

Figure S7: High-resolution ToF-SIMS ion images of microtome cut CuDEPP composite electrodes: (a) Pristine electrode measured in negative ion mode and (b) pristine electrode measured in positive ion mode. (c) Cycled electrode measured in negative ion mode and (d) cycled electrode measured in positive ion mode. For the pristine electrode, the current collector is shown in turquoise (Al signals) and the microscope slide in grey in form of silicon signals. For pristine and cycled electrodes, organic species are shown in blue and CuDEPP species in purple. Mg species were only detectable in cycled electrode and are shown in yellow. Organic species (blue): Sum of (C_x, C_xH_y, $C_xH_yO_z$, $C_xH_yN_z$, $C_nH_xN_yO_z$). CuDEPP species (purple): Sum of (CuC_xN_y, $CuC_xH_yN_z$, $CuC_xN_yO_z$). Cu & Mg species (yellow, negative polarity): Sum of (Cu_xCl_y, $Cu_xO_yF_z$, $Mg_xCu_yCl_z$, Mg_xF_y, Mg_xCl_y, $Mg_xF_yCl_z$). Mg species (yellow, positive polarity): Sum of (Mg_xF_y, Mg_xO_y, $Mg_xO_yH_z$). All measurements were conducted in delayed extraction with 30 keV Bi_3^+ as primary ion species.
